# MongoDB Database as Storage for GPON Frames

**DOI:** 10.3390/s20216208

**Published:** 2020-10-30

**Authors:** Martin Holik, Tomas Horvath, Vaclav Oujezsky, Petr Munster, Adrian Tomasov, Sobeslav Valach

**Affiliations:** Department of Telecommunication, Brno University of Technology, Technicka 12, 616 00 Brno, Czech Republic; horvath@feec.vutbr.cz (T.H.); oujezsky@feec.vutbr.cz (V.O.); munster@feec.vutbr.cz (P.M.); xtomas32@stud.feec.vutbr.cz (A.T.); valach@feec.vutbr.cz (S.V.)

**Keywords:** analysis, FPGA, GPON, MongoDB, storing

## Abstract

This work is focused on creating an open-source software-based solution for monitoring traffic transmitted through gigabit passive optical network. In this case, the data are captured by the field-programmable gate array (FPGA) card and reassembled using parsing software from a passive optical network built on the International Telecommunication Unit telecommunication section (ITU-T) G.984 gigabit-capable passive optical network GPON recommendation. Then, the captured frames are converted by suitable software into GPON frames, which will be further processed for analysis. Due to the high transfer rate of GPON recommendations, the work describes the issue of writing to the Mongo database system. In order to achieve the best possible results and minimal loss of transmitted frames, a series of tests were performed. The proposed test scenarios are based on different database writing approaches and are implemented in the Python and C# programming languages. Based on our results, it has been shown that the high processing speed is too high for Python processing. Critical operations must be implemented in the C# programming language. Due to rapid application development, Python can only be used for noncritical time-consuming data processing operations.

## 1. Introduction

Optical networks are divided into active optical networks (AONs) and passive optical networks (PONs). Active networks require some of the network elements to be powered and are used primarily in transport core networks. On the contrary, the elements of the passive network do not require any power supply and, thanks to their low purchase price, are used primarily as a so-called last mile solution to connect end users. They are also common in networks combining metallic and optical lines, which are created by the gradual replacement of metallic lines [1,2,3,4,5].

Currently, the most widespread International Telecommunication Union telecommunication section (ITU-T) recommendations include Gigabit-capable passive optical networks (GPON) and 10-Gigabit-capable passive optical network (XG-PON), which are based on time division multiplex (TDM) [6,7]. The size of the frames is therefore not exactly given but is limited by time periods of 125 μs. In the ascending direction, the concept of time division into 125 μs sections is retained, but it is supplemented by regular time ticks/marks, which are used for synchronization. To enable bidirectional communication, different wavelengths are used for uplink and downlink. For GPON, a signal with a wavelength of 1490 nm is used for the downlink and 1310 nm for the uplink and 1577 nm in the downlink and 1270 nm in the uplink direction for XG-PON [2,3,4,6,8,9,10,11].

The problem of data traffic analysis lies in the large flow of data that is transmitted through passive optical networks. This is an area of big data processing, where it is impossible to analyze communication in the usual way. Although there are tools for data analysis in a commercial environment, the problem of these tools is associated not only with a problematic and very expensive licensing policy but also with closed program code and the inability to use the interface for self-testing. Today’s analyzers are difficult to reach for small Internet services providers (ISPs) with hundreds of customers. Our solution is focused on providing a modular solution that should be distributed under open-source licenses. Our proposed solution is built with field-programmable gate array (FPGA) card and appropriate control software that will be installed on the operating system.

The main contribution of the article is the experimental verification of the write speed of traffic transmitted in the downstream direction of the passive optical network. This work is focused on traffic from the GPON network, but later it is planned to create a version for the analysis of XG-PON networks. After the successful storage of all important data, the data will be further processed in order to detect hidden security threats using artificial intelligence.

The rest of this paper is structured as follows. Section 2 provides an overview of the related works. Section 3 describes the necessary prerequisites that are required for testing including a data structure built on Mongo database. Section 4 is focused on the description of test scenarios. The individual scenarios are divided according to the way in which GTC frames are written to the repository and according to the programming languages used. Section 5 provides a discussion of the results. Finally, Section 6 concludes the paper.

## 2. Related Works

In recent years, several PON-related studies have been published. The authors in [12] describe a novel method for low-latency 10-Gigabit-capable symmetric passive optical network (XGS-PON) mobile front-haul for small cell in cloud radio access network (C-RAN) based on traffic estimation. They proposed the adaptive-Learning dynamic bandwidth allocation (DBA), which reduces jitter in optical distribution network (ODN), packet loss ratio, delay, and increasing utilization performance. The article [13] deals with the impact of report message scheduling (RMS) in 1/10 G passive optical networks. The researchers reduce the idle time of channel during a reporting optical network unit (ONU) buffer occupation for data transmission. The work [14] also deals with a dynamic bandwidth allocation algorithm for long-reach systems. The primary purpose of this research is to reduce inefficiency occurs in the DBA upstream protocols because of the large propagation time between optical line termination (OLT) and ONUs. This algorithm is independent of the SI and uses real data queuing information without prediction. In [15] the authors present a novel opportunity for data transmission in GPON with sensing data encapsulation. The sensory system uses different wavelengths; the results are transmitted in GPON encapsulation method (GEM) frames.

Another research field in passive optical network is their software defined controlling. In general, software defined networks (SDNs) are a very popular and promising solution in metro and core networks; the software controlling can be applied in access networks [16,17,18,19,20,21,22,23]. A big network with multiple OLTs will require a complex support of technician; however, the SDN controlling with one central point may help to make a management easier. Multi-OLT networks have to control DBA algorithms to ensure the quality of service (QoS) [24,25,26].

A data analysis is not a trivial task in passive optical networks defined by ITU recommendations. First of all, all data are encapsulated into GPON transmission convergence (GTCs). The structure of GTC is completely different in comparison with Ethernet frame; however, Ethernet frames are encapsulated into them. In recently years, many publications have been published about simulations of data traffic in passive optical networks [27,28,29]. Note that each simulation tool supports physical or above layers; this means that the physical parameters will be evaluated or data transmission will occur separately. Nowadays, the available tools for data analysis in passive optical networks are in stock but with one main limitation—postprocessing data representation [30,31].

Analyzing the transmission convergence layer of passive optical networks is not a new problem. Several patents in the field of passive optical network analysis have been published in the past. Still, current developments in technology and world affairs place greater emphasis on detecting unwanted processes that could cause the leakage of sensitive information. The work [32] discloses a method of reducing a PON network’s energy consumption by monitoring the physical layer and the GTC layer. In the work [33], the authors proposed a GPON network extender built on an FPGA array. For this extender to work properly, adding a sync bit sequence to the optical layer during coarse wavelength division multiplexing (CWDM) transmission was necessary. The designed extender with OLT unit allows them to connect up to 512 client devices but changes the activation process of ONU units according to ITU-T recommendation [3] were necessary. The article [34] also deals with the issue of security using FPGA arrays. The authors created an advanced encryption standard (AES) encryption algorithm for hardware transmission encryption using the FPGA card. As part of the Fabulous EU Strep project, the authors dealt with the simulation of traffic transmission and its digital processing using algorithms in FPGA arrays [35].

Previously mentioned works, focused on FPGA card data processing, are primarily focused on increasing the quality of operation of passive optical networks or security from the perspective of third parties, for example, against eavesdropping. The focus of our research is to analyze the communication itself against unwanted processes that are not defined in the ITU-T recommendations of GPON networks. Currently, the market offers many devices of different manufacturers that are not compatible with each other due to various implementations of optional values or specification in their recommendations. Mutual incompatibility and the use of one manufacturer’s equipment on a single network could be a hidden threat to sensitive data leakage. This research should make it possible to verify that the data transmitted follow the ITU-T recommendations.

## 3. Prerequisites

**Figure 1:**
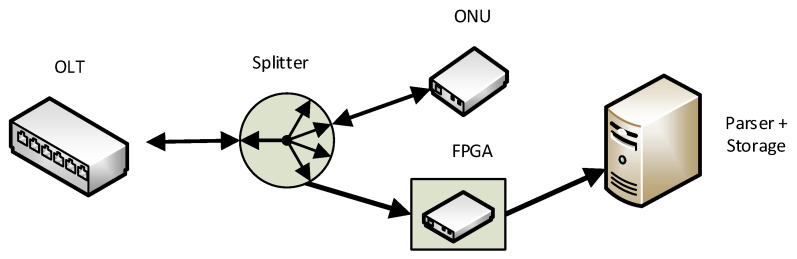
Scheme of our measurement topology.

Our test scenario was designed with regard to the requirements for capturing traffic from passive optical networks. The entire circuit diagram of the topology of the currently used optical network is shown in Figure 1. The FPGA module developed by our partner is connected to the passive optical network, which captures the transmitted bits in the downlink direction, i.e., from the OLT unit to the ONU unit [36]. The captured data are reassembled into higher layer frames and stored in a folded form. Depending on the recommendation used, it is necessary to perform the optimal design of the data structure for the maximum speed of writing data to the storage. The second, but less critical, operation is the optimization of the output for future reading and subsequent data processing.

**Figure 2:**
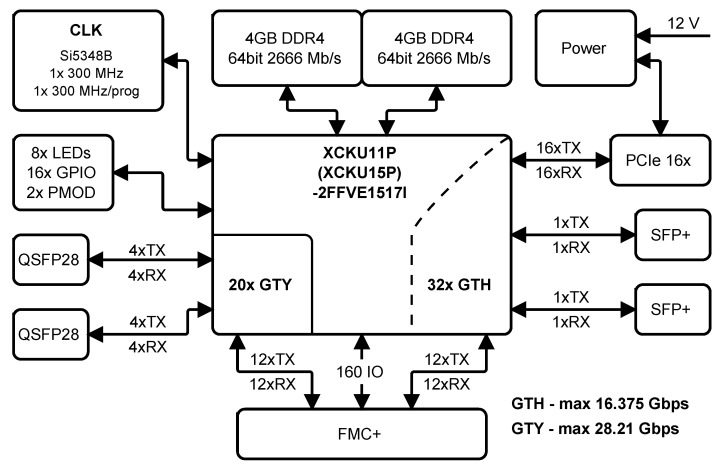
The block diagram of developed field-programmable gate array (FPGA) card.

**Figure 3:**
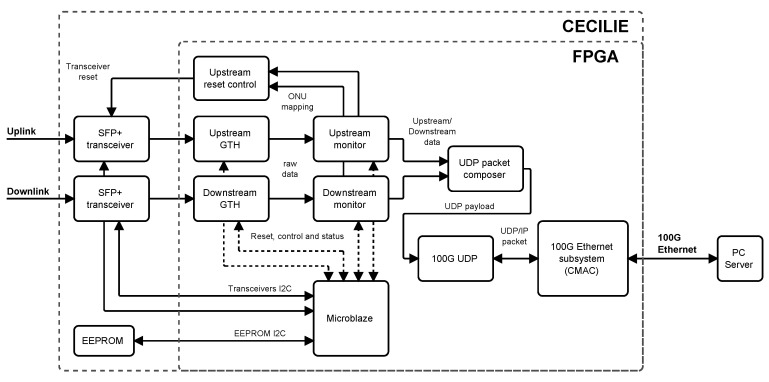
The internal configuration of Cecilia card.

The FPGA card (named Cecilia) is specially designed for processing and transferring data streams from the GPON and Ethernet interfaces up to 100 Gbit data rates. The card is equipped by a middle class Xilinx’s Kintex UltraScale + FPGA—XCKU11/15P-2FFVE1517. The FPGA provides 20 pairs of gigabit transceiver Y (GTY) transceiver operating up to 28.21 Gbit/s and 32 pairs of gigabit transceiver H (GTH) transceiver with maximum achievable bit rate 16.375 Gbit/s [37]. Figure 2 depicts a block drawing showing card configuration and Figure 3 shows the main peripherals.

This parsing software utilizes one GTH transceiver connected to the small form-factor pluggable (SFP+) cages. One cage is used for upstream and the second one for downstream monitoring and stream capturing. The captured data are transferred directly into a host computer main memory by peripheral component interconnect express (PCIe) interface. Due to a low rate stream, the PCIe can be configured as x4 interface to save FPGA resources and lower power consumption.

A firmware implementation is divided into three blocks. The first block captures data from downstream and upstream links and provides raw data to the monitoring block handling ONU’s mapping and upstream reset generation. The downstream capture unit oversamples input stream by ratio of five and digitally recovers incoming data.

The time stamped data are sent through the PCIe subsystem to the computer host main memory. The logic utilization is 9.5% of the XCKU11P device without any special optimization technique. The design includes debug support features consuming more than 50% of overall application.

In general, this type of application can be processed by application-specific integrated circuits (ASICs) or Network processors. The FPGA is more flexible and gives us a high level of freedom in development of new monitoring techniques and ideas.

The Cecilia card allows two connection modes. The first mode preverification (PV) can be used as a standalone solution and it is connected to the server that processes the data via a 10 gigabit Ethernet port. The second option is to use the card as a server solution when inserted into the PCIe slot of the server on which the control software is running. During our tests of the FPGA card, a stand-alone connection was used. The whole concept of data capture consists of four layers. The first and, at the same time, the lowest layer is the FPGA card itself, which sends via the Ethernet interface to the server, where they are captured by the listening module. The intercepted data is processed at the application module level by parsing software written in the C# programming language, allowing data to be stored in a repository for GPON frames. The stored data is ready for processing or can be viewed using a web interface written in the Python programming language [36,38,39]. Our solution does not include a step to create COM-callable wrapper and call using Python’s component object model/object linking and embedding (COM/OLE) from the complexity perspective.

During the tests, a passive optical network based on the ITU-T G.984 GPON recommendation was used, which offers a downstream transmission speed of 2.488 Gb/s [8]. The total number of GEM frames per second is very difficult to estimate because it is a problem to estimate the correct number of frames with relevant data. However, it is relatively easy to determine the lower limit. A GEM frame can carry a maximum of 4095 bytes of data, and therefore a minimum of about 76,000 GEM frames per second can be expected. Assuming that one GEM frame carries half the maximum size, the storage must handle twice, that is, 152 thousand GEM frames per second. All transmitted GEM frames are encapsulated as user data into 8000 GPON frames.

## 4. Testing Scenarios

The first data storage tests were performed using Microsoft structured query language (MS SQL). MS SQL is a database system designed for a large volume of data, and a suitable design can divide the data into tables to avoid duplicate records [38,39,40]. Better results were obtained using the MongoDB document database system. This system’s advantages include native data storage in JavaScript object notation (JSON) format, which is more advantageous not only for easier and faster writing to the database but also for the reading speed, which is often higher than the MS SQL database. The second advantage of using a Mongo database was eliminating problematic data conversion, which caused up to 90 percent loss of reshaped frames in the previous case.

**Figure 4:**
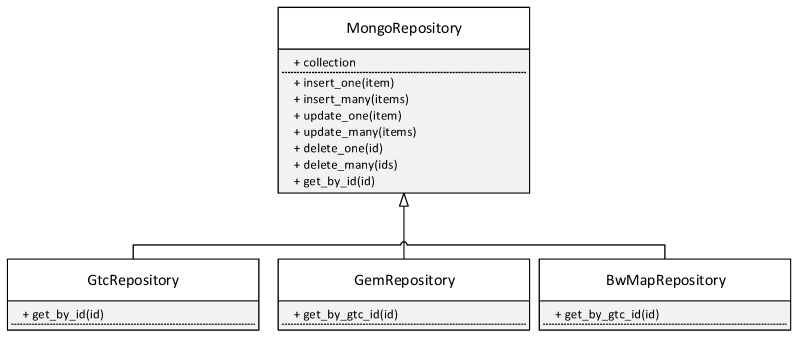
UML diagram—MongoRepository.

The Mongo database is supported by many programming languages, including Python and C#, with which we performed write testing. The MongoRepository class has implemented methods for testing all tested scenarios, as shown in the unified modeling language (UML) diagram in Figure 4. This class is used as a general model and for GEM frames, GPON frames, bandwidth maps (BWmaps), GemRepository, GtcRepository, and BwMapRepository. Each class represents one collection in the Mongo database. The data were not intentionally divided into smaller collections, not only due to the possible slowdown in the writing speed, but also the complexity of the logic that would put the data back into the final frames. The UML diagram is only a general design. The individual naming of methods in the UML diagram is adapted to the naming conventions of the programming language used.

Individual frames are stored as Mongo database documents and as a whole form a collection. Information about the smallest unit of the GPON frame is stored in individual documents, i.e., a GPON header without a BWmap field, a BWmap field and all GEM frames transmitted in the body of the GPON frame. The data is stored in binary javascript object notation (BSON) format, which is convenient for further processing. The proposed structure in the Mongo database allows to store data in inconsistent or nonstandard form against relational databases, which can be especially useful when storing undefined traffic compared to the G.984 recommendation.

To achieve the most realistic simulation of real operation, each collection (GTC, GEM, BWmap) in the database was written for each scenario. GTC documents and bandwidth maps are connected by a direct link (1:1), while multiple links (1:N) are proposed between GTC and GEM documents. From the above, it is clear that the GEM of documents will be many times more and therefore the speed of writing will be limited by writing GEM documents.

The test data set is the same for both languages and is based on the data actually captured by the FPGA card. In order to make the operation uniform during the test and to be able to realistically compare the results between the individual test scenarios, one GPON frame is selected from the real operation. This GPON framework is periodically generated by a script in a given language and sent for storing in the database.

The test scenarios were run on a computer station with the same configuration as shown in Table 1.

### 4.1. Serial Data Writing

Serial writing method is a way in which individual data are written sequentially. Due to the proposed structure of the Mongo database, this is a way in which the GPON header of the frame is written first, including both payload length indicator (Plend) fields but without the BWmap field. After writing the header, the BWmap field is written and finally the document containing the GEM frames.

### 4.2. Mass (Batch) Data Writing

There are two ways to test batch enrollment. As in the previous case, the GPON header of the frame without the BWmap field is written first. The remaining parts of the GPON frame are stored in a field in the writing application and linked to the document key of the specific GPON frame, which was obtained after writing the specific GPON frame header to the Mongo database. Two batches are stored in the repository, the first with BWmap fields and the second with individual GEM frames.

The second way of writing is very similar. The principle of writing is the same, differing only in the amount of data that is written in the batch. In contrast to the previous method, where all BWmap fields and GEM frames were written at once for the whole batch of GPON frames, the write is always related to one GPON frame.

### 4.3. Asynchronous Writing Method

**Figure 5:**
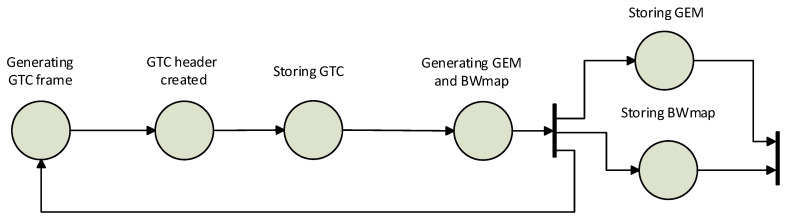
Asynchronous writing method.

The asynchronous writing method is based on writing after the previous method, i.e., after GTC frames. In this test, the emphasis is on parallelism. Unlike the previous methods, each write waited for its result, while the asynchronous write waited for the result at the end of the test. Better results should be obtained by using asynchronous code. The principle of writing is shown in Figure 5.

### 4.4. Asynchronous Bulk Write with Parallel Generation

**Figure 6:**
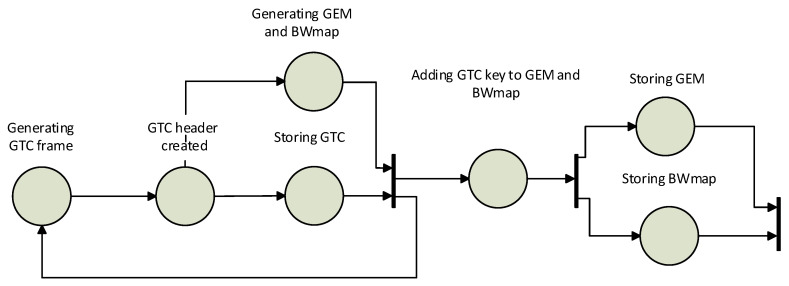
Asynchronous bulk write with parallel generation.

This test scenario is again a modification of the previous scenario. The only difference is when BWmap fields and GEM frames are written. The previous scenario saved the GTC header, when the unique key of the document was returned, then the GEM header of the frame and the BWmap field were generated, which were then stored asynchronously. In this scenario, parallelization is already achieved when saving the GPON frame. While waiting for the document’s GTC key to be obtained, a BWmap and GEM frame field is generated. While the key is being added to the second generated data, the process of generating another GPON frame is already started. Detailed process flow is shown in Figure 6.

## 5. Results

**Figure 7:**
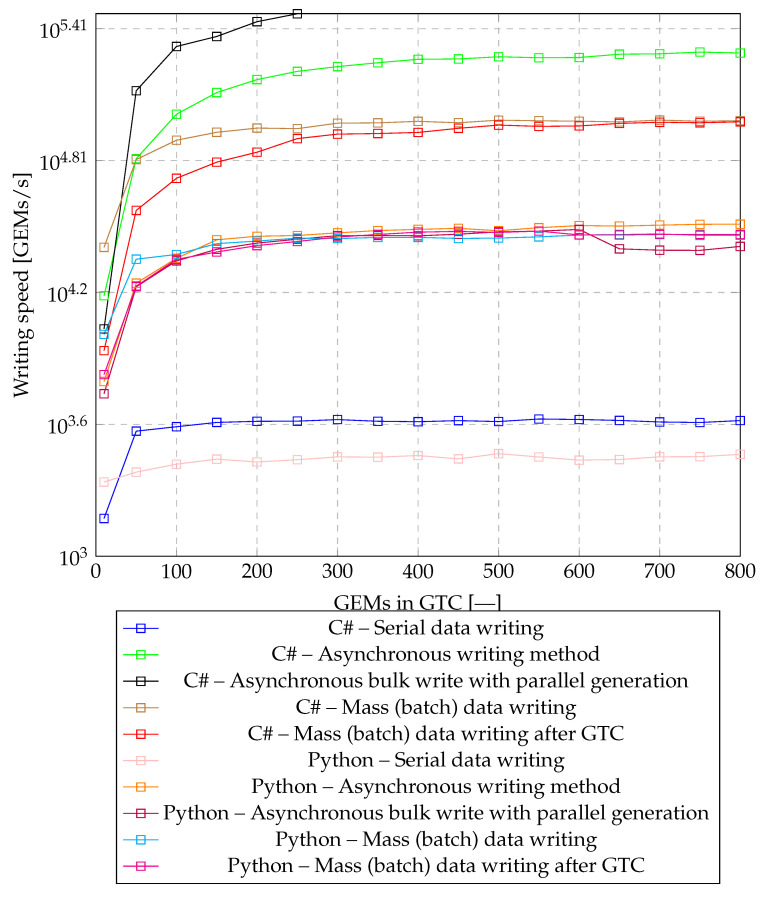
Graph of dependence of writing speed based on the used writing technique.

Serial write speeds for Python ranged from approximately 2100 to 2900 GEM documents per second. In most cases, C# achieved faster write speeds ranging from 1500 to 4100 documents per second. The dependence of the write speed on the number of GEM documents can be seen in Figure 7. Serial writing does not seem appropriate, because its speed is many times less than the minimum required.

The bulk write speed ranged from 6000 to 32,000 GEM headers per second for Python and from approximately 8500 to 97,000 GEM headers per second for C#. As can be seen from Figure 7, the bulk writing of all GEM documents is faster for smaller numbers of GEMs. At more than 250 GEM, the performance difference begins to decrease and is almost zero for 800 elements.

The bulk write speed ranged from 6000 to 32,000 GEM headers per second for Python and from approximately 10000 to 325,000 GEM headers per second for C#. Asynchronous storage in Python provided almost no acceleration compared to bulk writing. Performance testing in C# shows that asynchronous access is not suitable for small numbers of GEM headers up to 50, where the write speed is slower than for bulk write. However, for larger quantities, there is a significant increase in performance, with both the minimum and expected write speeds achieved in both scenarios. An hybrid solution (precompiled C# package) might solve the issue.

The results obtained, on which the above graphs are based, are also introduced in Table 2. The complete testing differed based on the change in the number of GEM frames encapsulated in GPON frames. During testing, results were measured for 10, 50, 100, 150, 200, 250, 300, 350, 400, 450, 500, 550, 600, 650, 700, 750, 800 GEM frames encapsulated in GPON frames. Due to the high number of measured values, only a subset of them was listed in the table.

## 6. Discussion

The results of this work are based on previous experiments with the MS SQL database, described in the publications [38,39,40]. The main requirement for future work was to optimize the writing speed as much as possible and also to enable easy data acquisition, which may not comply with G.984 recommendations. Based on these requirements, the Mongo database system was selected and a series of experiments to write to the database were performed. Because these are high data rates, we consider working with data to be a big data area. The experiments performed were to show whether it is possible to reach the minimum writing speed with the selected area. Two programming languages were used during the experiments. The Python programming language was chosen as the environment for rapid application development and the language that will be used for future processing, for example using analysis in TensorFlow. The second programming language that is used to implement test scenarios is C#. The parsing software, described in publication [38], is implemented in this programming language.

After initial examination of the writing possibilities, it was quite clear that the serial writing would be completely unsuitable for the described bit rate, but it was mentioned for interest. The aim of the established tests was to parallelize the writing of data to the database as much as possible and thus achieve maximum writing speed. From the achieved values in Table 2, it is clear that the Python programming language is very slow and therefore unsuitable for very fast operations. During tests with the C# programming language, it has been shown that sufficient speed can be achieved to write data fast enough with zero GPON frame rate.

The proposed experiments aimed to show the possibility of writing data from passive optical networks based on the G.984 GPON standard, which are currently still used in Europe. Due to newer types of passive optical networks (e.g., XG-PON), these tests can be used for testing if the input GPON frames are converted to XG-PON frames. By converting to a newer type of passive optical networks, the speed limits of both the programming language and the data storage itself will probably be reached and it will be necessary to adjust the whole principle of frame storage.

## 7. Conclusions

This paper summarizes the issue of recording traffic from a passive optical network to a repository built on the popular Mongo document database. The main goal of this work was to select a suitable way for data to be stored in the repository. Based on the performed testing, it is possible to use the Mongo database to store such a large amount of data, but even so, values at the limit of the required standards are achieved. If we wanted to apply current methods for storing traffic from faster networks (such as XG-PON), it would be necessary to use other technologies such as Apache Kafka. The stored data can also be processed by the tested Python language, because there is no longer a speed-limiting element. The data stored in the repository will be used for the following analysis of the operation, for example using artificial intelligence.

Future work will be focused on the analysis of stored data, design of methods for traffic analysis and modification of the presented solution for use in passive optical networks with higher data transmission. In addition to the ability to use other technology, processing speed can be increased by adjustment of the computer configuration. Multiprocessing might be used to increase the parallelization of computational operations to achieve better results.

The second option is to increase the number of solid-state drives (SSDs) and create a raid array. Another thread of this work could be focused on the data preprocessing performance directly on the FPGA card. Which could, after appropriate consideration, filter, ignore, or delete unnecessary data. For example, the same repeated physical synchronization (Psync) fields can be ignored. They indicate only the beginning of the GPON frame.

The last option to achieve better write results, which can also be a big challenge, is to send data directly to the Mongo database from the FPGA card. Artificial intelligence neural networks appear to be suitable methods for analysis.

## Figures and Tables

**Table 1:** Hardware used for testing.

Component	Caption	Key Parameters
CPU	AMD Ryzen 7 2700X	3.2 GHz, 8 physical cores
RAM	HyperX Predator 4 × 8 GB	32 GB DDR4 3333 MHz
HDD	M.2 SSD WD SN500	Writing 1450 MB/s

**Table 2:** Reached results from writing tests.

GEMs in GTCs	10	100	200	300	400	500	600	700	800
C# – serial	1488	3903	4131	4206	4114	4121	4211	4102	4163
C#–batch all	25,707	79,302	90,050	94,817	96,642	97,867	96,790	97,943	97,312
C#–batch GTC	15,456	104,167	149,925	171,821	185,529	190,549	189,095	196,574	198,265
C#–asynchronous GTC	8681	53,135	69,881	84,507	86,003	92,920	92,061	95,720	96,165
C#–parallel generating	10,905	212,314	275,482	313,152	325,468	314,861	313,972	316,170	312,744
Python–serial	2182	2632	2693	2842	2883	2940	2747	2844	2919
Python–batch all	10,300	23,856	27,466	28,198	28,557	28,341	29,254	29,617	29,168
Python–batch GTC	6751	22,655	26,188	28,655	30,161	30,075	29,299	29,435	29,439
Python–asynchronous GTC	6278	22,915	28,893	29,943	31,052	30,612	32,316	32,466	32,784
Python–parallel generating	5520	22,301	26,893	29,071	29,022	30,257	31,028	24,946	25,959

## Abbreviations

The following abbreviations are used in this manuscript:

AES	Advanced encryption standard
AON	Active optical network
ASIC	Application-specific integrated circuit
BSON	Binary JSON
BWmap	Bandwidth map
C-RAN	Cloud radio access network
COM	Component object model
CPU	Central processing unit
CWDM	Coarse wavelength division multiplexing
DBA	Dynamic bandwidth algorithm
FPGA	Field-programmable gate array
GEM	GPON encapsulation method
GPON	Gigabit-capable passive optical network
GTC	GPON Transmission Convergence
GTH	Gigabit transceiver H
GTY	Gigabit transceiver Y
HDD	Hard disk drive
ISP	Internet services provider
ITU-T	International Telecommunication Unit telecommunication section
JSON	JavaScript object notation
MS SQL	Microsoft structured query language
ODN	Optical distribution network
OLE	Object linking and embedding
ONU	Optical network unit
OLT	Optical line termination
PCIe	Peripheral Component Interconnect Express
Plend	Payload length indicator
PON	Passive optical network
Psync	Physical synchronization
PV	Preverification
QoS	Quality of Service
RAM	Random access memory
RMS	Report message scheduling
SDN	Software defined network
SFP	Small form-factor pluggable
SSD	Solid-state drive
TDM	Time division multiplex
UML	Unified modeling language
XG-PON	10Gigabit-capable passive optical network
XGS-PON	10-Gigabit-capable symmetric passive optical network
